# Printed Models for Better Prediction of Surgery in Patients with Double Outlet Right Ventricle

**DOI:** 10.1007/s00246-024-03747-8

**Published:** 2025-01-06

**Authors:** Sterre F. Hoogerbeets, Arno A. W. Roest, Israel Valverde, Gorka Gomez-Ciriza, Lucia Kroft, Mark G. Hazekamp

**Affiliations:** 1 Dept. Cardiothoracic Surgery, Leiden University Medical Center, Leiden, The Netherlands; 2 Dept. Pediatric Cardiology, Leiden University Medical Center, Leiden, The Netherlands; 3 Division of Pediatric Cardiology, The Labatt Family Heart Centre, Department of Pediatrics, The Hospital for Sick Children, University of Toronto, 555 University Ave, Toronto, ON M5G 1X8 Canada; 4 Hospital Universitario Virgen del Rocío, HUVR Technological Innovation Group, Industrial Engineering, Seville, Spain; 5 Dept. Radiology, Leiden University Medical Center, Leiden, The Netherlands; 6 Utrecht, The Netherlands

**Keywords:** cDORV, Biventricular, Congenital heart disease, Congenital heart surgery, Imaging, Pediatric, Preoperative care

## Abstract

The complex and variable anatomy of complex double outlet right ventricle makes it imperative to understand the spatial anatomic structures to determine whether it is feasible to repair the anomaly in a biventricular or univentricular fashion. Biventricular repair should be aimed for but is not always feasible. Choosing the correct surgical technique is of great importance in surgical planning of biventricular repair. Conventional imaging is typically insufficient to predict feasibility and technique of biventricular repair. The gap between virtual images and spatial reality can be filled using 3D prints. Retrospective observational study of all available imaging including 3D prints and operative reports in 13 patients. 3D-prints enabled accurate prediction of biventricular repair in 8 cases and of univentricular repair in 2 cases. In 2 patients, no precise prediction was possible. One 3D-print was created post repair. 3D-prints accurately predicted the optimal technique for achieving biventricular repair in 8 cases. Conventional imaging could not accurately predict biventricular repair of optimal surgical technique in any patient. In complex double outlet right ventricle, 3D-printing can predict feasibility of biventricular repair better than conventional imaging. Additionally, 3D-prints can predict the type of surgical technique better. 3D-printing is also helpful in preoperative discussion with parents, caretakers, and pediatric cardiologists. 3D-prints are strongly recommended in patients with complex double outlet right ventricle.

**Graphical Abstract:**

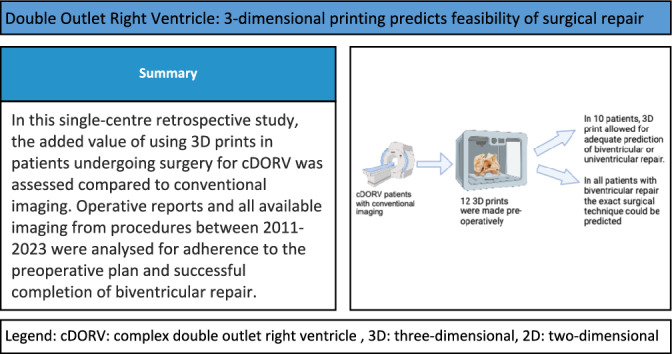

## Introduction

Double Outlet Right Ventricle (DORV) is a form of ventriculoarterial connection where the aorta and pulmonary artery arise completely or predominantly from the right ventricle [1]. There is a vast variety of different morphological features and clinical presentations of DORV [2]. DORV lies on a spectrum where on one side it shares the anatomic features of tetralogy of Fallot [3]. On the other side it shares the anatomic traits of transposition of the great arteries (TGA) with a ventricular septal defect (VSD) [3]. A VSD is present in all cases of DORV and is an essential feature of the malformation [4]. Taussig–Bing anomaly was first described in 1949 as a combination of TGA, DORV and subpulmonary VSD [3, 5].

Complex DORV (cDORV) is here described as a form of DORV that is different from Tetralogy of Fallot or Taussig–Bing anomaly.

Because of the complex and variable anatomy of DORV patients, it is imperative to understand the spatial anatomical structures to determine surgical approach [6]. An important consideration for surgical planning is whether it is feasible to repair the cDORV anomaly in a biventricular way. If biventricular repair is not feasible an univentricular pathway must be followed [1]. While biventricular should be aimed for, it is not always obtainable [1]. Choosing for biventricular repair when this is not possible may result in serious complications. However, unnecessary univentricular repair carries all the disadvantages of the Fontan circulation [1]. This highlights the importance of carefully choosing between biventricular or univentricular repair before surgery.

Furthermore, choosing the correct surgical technique is of great importance in surgical planning of cDORV. Conventional imaging techniques (echocardiography, angiography, CT or MRI) are frequently insufficient to predict biventricular repair and plan the surgical technique accordingly. Conventional 2D imaging can determine the diagnosis and status of the ventricles [7]. CT and MRI provide 3D data but may result in wrong interpretations as it is shown on a 2 D computer screen [1]. This creates a gap between virtual images and the spatial reality. This gap can be filled using 3D prints [8]. 3D techniques allows digital files to be converted into 3D hollow objects [1]. 3D models are reported to allow for a better understanding of the heart’s spatial anatomy and eliminate miscommunication among imagers and surgeons [1]. 3D printing has been used in other cardiothoracic anomalies such as aortic arch malformations [9, 10]. 3D prints can also be used to tailor the surgical approach toward the specific anatomy of the patient [1]. 3D prints will allow for better consultation with the family/caretakers before and after surgery. Furthermore, physical prints allow physicians with less expertise in the interpretation and analysis of cardiovascular images to get a good understanding of spatial cardiac relationships [11].

When two well-developed ventricles are present, biventricular repair should be aimed for. The LV is then connected to either the aorta or the pulmonary trunk (and then combined with an arterial switch operation (ASO)) [12]. Enlargement of the VSD is sometimes necessary to create an unobstructed LVOT [12].

Factors that should be taken into account are a possible loss of RV volume, whether there is sufficient space between the tricuspid valve and pulmonary valve and the absence of straddling of tricuspid valve chords. [1] Assessing all latter factors can be challenging in conventional 2D imaging. 3D prints better determine the possibility to create an adequate baffle in pre-surgical planning [7].

In this article, our experience with assessing the feasibility of biventricular repair and predicting the best surgical technique using 3D prints in cDORV is analyzed in thirteen non- consecutive (patients without a 3D print are not included) patients. We do this by conducting a single-center retrospective study of operative reports and all available imaging including the 3D-printed hearts procedure from 2011 to 2023.

## Patients and Methods

### Ethics Statement

A waiver from the local medical ethical committee was obtained.

### Patients

Thirteen patients are enrolled in this study, who had their definite procedure from 2011 to 2023.

The non-consecutively included cDORV patients with 3D prints had a median age of 13.5 months (IQR = 3.0–48 months) during the definite procedure. As shown in Table 1, 9 patients are diagnosed with TGA; 3 patients had normal relationship of the arteries (NRA); and in one patient the aorta was left to the pulmonary artery. As seen in Table 5 in Appendix 1, 11 patients underwent palliative surgeries before the definite biventricular or univentricular procedure: six patients received a Pulmonary Artery Banding (PAB), 3 patients received Bidirectional Cavopulmonary Anastomosis (BCPA), and 4 patients received one or more Modified Black Taussig Shunts (MBTS).

**Table 1:** Patient characteristics obtained from 3D print important for surgical planning

Pt nr	Age definite procedure (months)	Great arterial relationship	Location VSD	Pulmonary stenosis	Coronary anatomy	Straddling	Ventricular hypoplasia	Aortic abnormalities	Other anomalies
1	62	TGA	Non-committed	No	2LCXR	No	–	–	–
2	32	NRA	Multiple	No	1R2LCX	No	LV hypoplasia	Coarctation	–
3	17	TGA	Multiple	No	1RLCX	No	–	–	–
4	81	Ao left to PA	Inlet	No	2RLCX	Straddling TV	–	–	Dextrocardia, AV discordance
5	55	TGA	Multiple	Yes	1R2LCX	Straddling TV	–	Aortic Arch obstruction	Double VCS, juxtaposition auricles, criss-cross AV-connections
6	3	TGA	Non-committed	No	1L2CXR	No	–	–	–
7	20	NRA	Non-committed	No	1R2LCX	No	–	–	–
8	8	NRA	Subaortic	Yes	2LCXR	No	–	–	Left isomerism, vena azygos continuation of VCI
9	10	TGA	Subpulmonary	No	1L2R	No	–	–	–
10	2	TGA	Outlet	No	1R2LCX	Straddling TV	–	–	–
11	9	TGA	Non-commited	No	1R2LCX	No	RV hypoplasia	–	–
12	3	TGA	Subpulmonary	Subvalvar	1L2R	No	–	–	–
13	13	TGA	Non-committed	Yes	1R2LCX	No	–	–	Vena azygos continuation of VCI, juxtaposition auricles

*Ao* Aorta, *AV* Atrioventricular, *BCPA* Bidirectional Cavo Pulmonary Anastomosis, *DORV* Double Outlet Right Ventricle, *LV* Left Ventricle, *MBTS* Modified Black Taussig Shunt, *NRA* Normally Related Arteries, *PA* Pulmonary artery, *PAB* Pulmonary Artery Banding, *PS* Pulmonary Stenosis, *RV* Right Ventricle, *TGA* Transposition of the Great Arteries (‘TGA position’), *TV* Tricuspid Valve, *VCI* Vena Cava Inferior, *VCS* Vena Cava Superior, *VSD* Ventricular Septal Defect

### Conventional Diagnostic Technique

All CT scans were performed on a 320-detector row CT scanner (Toshiba AquilionOne, Otawara, Japan) using the pediatric setting which is according to body weight [6]. All CT scans (n = 13) had adequate image quality and allowed for image segmentation for the 3D print. Echo, angiography, and CT scans were used to compare the feasibility of predicting surgical technique to 3D prints.

### Three-Dimensional Prints

We have designed and printed heart models since 2011. Our protocol for design and fabrication of a patient-specific 3D-printed heart model has been previously described in the literature [13–17].

Image segmentation was based on the previously acquired CT scans using ITK snap [18]. User-guided 3D active contour segmentation of anatomic structures significantly improved efficiency and reliability. Neuroimage 2006; 31:1116–28.] software was used by a consultant CHD cardiologist with more than 10 years expertise in cardiac imaging. The segmented geometry was exported as a 3D surface file into Meshmixer version 11.0.544 (Autodesk Inc., San Rafael, CA, USA) for computer-aided design. A 0.8-mm outer shell was added outside of the cardiovascular structures interface. The geometry was processed by Cura version 15.02 (Ultimaker BV, Netherlands) and sent to the 3D printer (BQ Witbox, Spain). All models were fabricated in polyurethane filament by fused deposition modeling. All heart models were able to be cut open and evaluated and most were printed as soft plastic, and some were made from harder plastic but no difference in quality of evaluation was seen depending on the material.

### 3D Print Evaluation

**Fig. 1:**
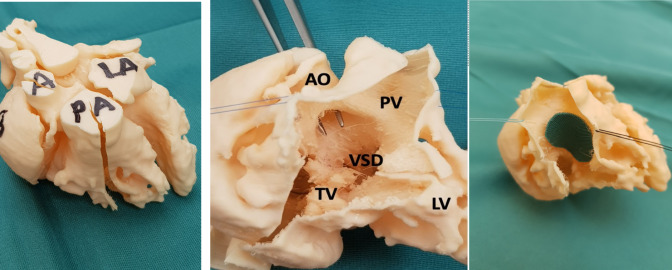
Three-dimensional prints used for determining surgical approach and technique. Own photographs that have not been previously published. Picture on the right shows LV to AO patch

**Fig. 2:**
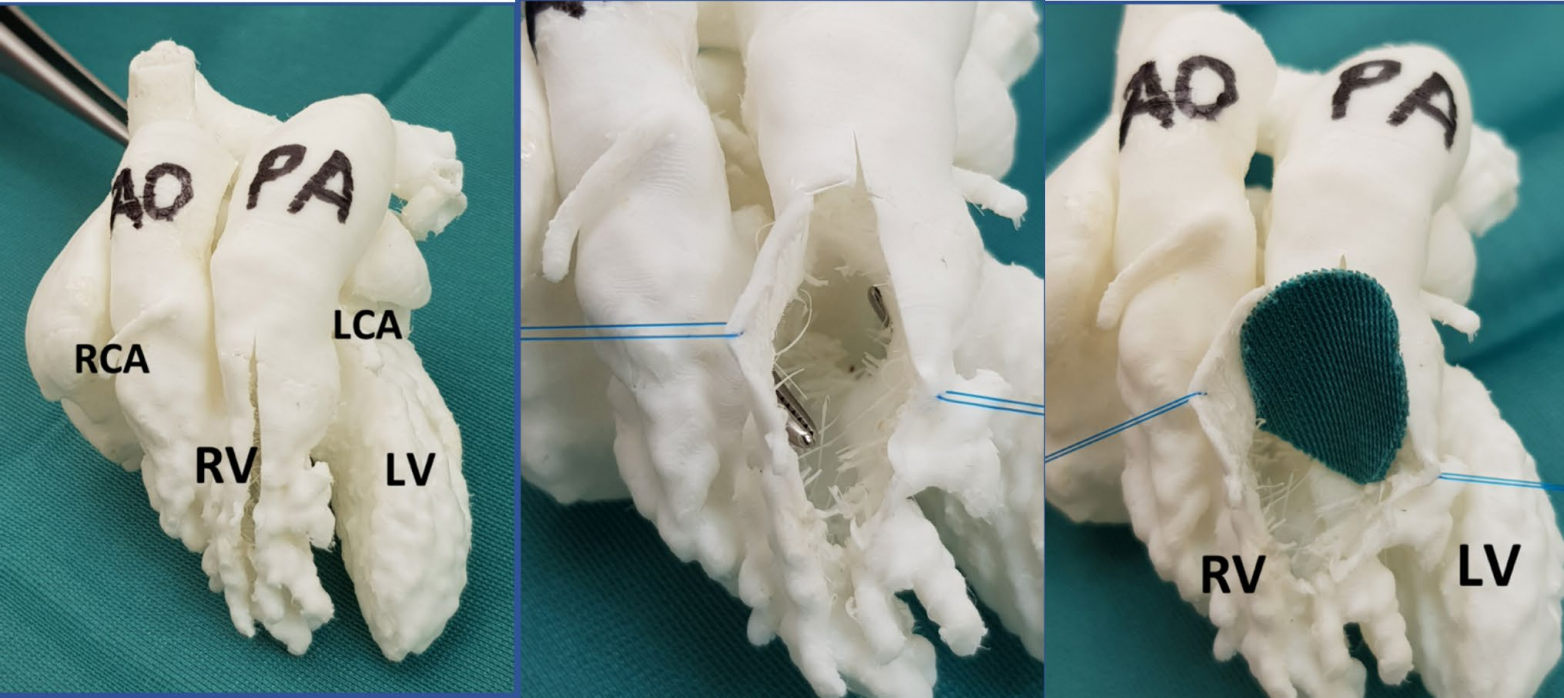
Three-dimensional prints used for determining surgical approach and technique. Own photographs that have not been previously published. Picture on the right shows biventricular repair at age of 6 weeks, LV to PA tunnel (via RV-tomy)

**Fig. 3:**
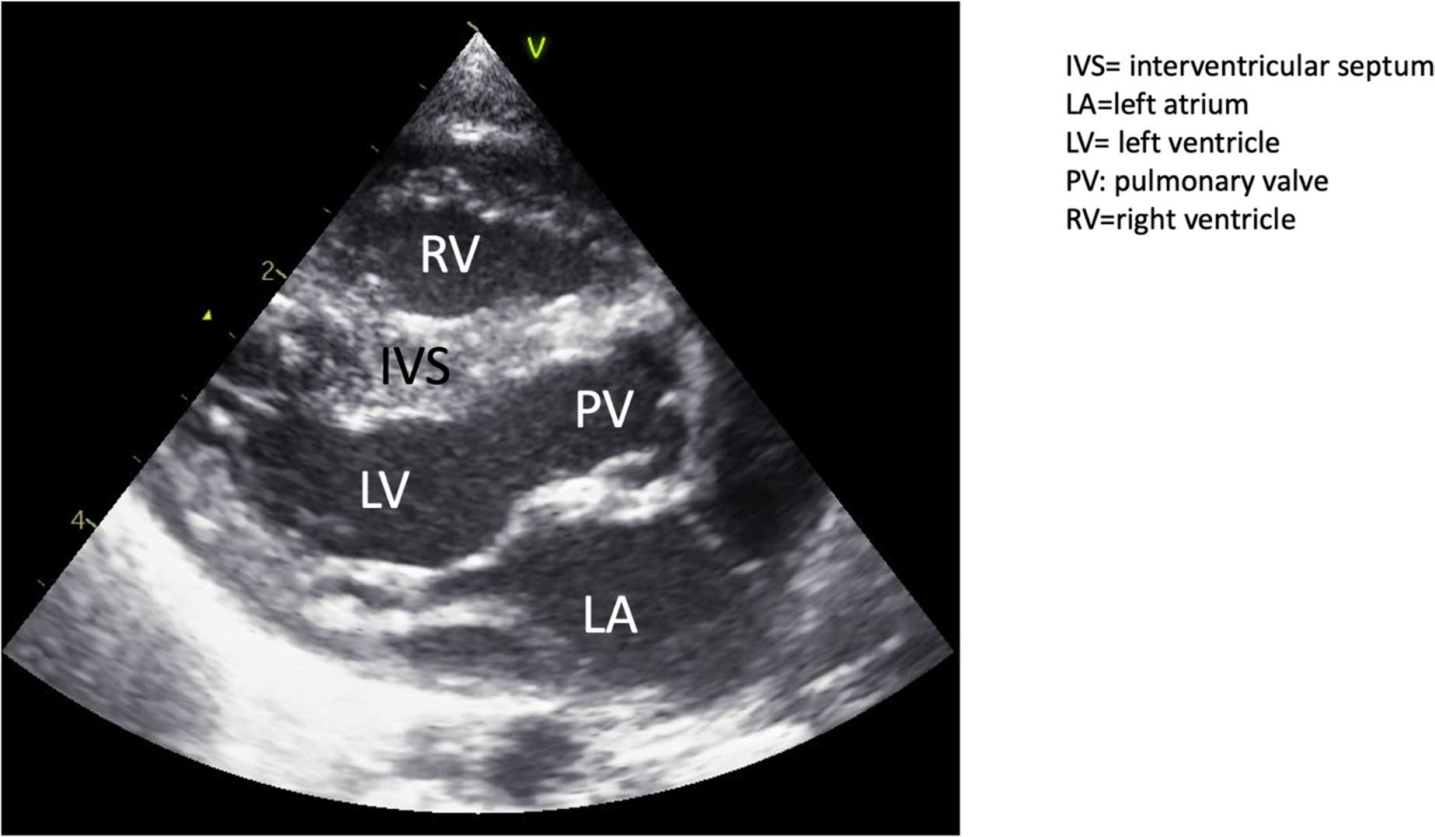
Echocardiographic long-axis view with no VSD visible

All diagnostic imaging were evaluated retrospectively by both a pediatric cardiac surgeon and a pediatric cardiologist. In Table 1, we evaluated all the different diagnostic aspects and anatomic features of the included patients (n = 13) (see Appendix 2). Figure 1 and Fig. 2 shows examples of 3D prints used to assess surgical procedure. In Fig. 3, an echo image is seen from a patient where there was no clear visible VSD while the LV is visible. This was however seen with a 3D model which demonstrated that this DORV could be septated to a biventricular heart after arterial switch and VSD closure.

### Data Availability

All relevant data are within the manuscript and its Supporting Information files.

Table 1 and 5 in Appendix showing patient characteristics and previous palliative surgeries.

Patient characteristics obtained from 3D print important for surgical planning.

## Results

### Considerations Based on 3D Print per Patient

The considerations based on the 3D print per patient were written out fully and can be found in appendix 3.

### Final Procedure

In total, 5 patients underwent the procedure with univentricular approach, and 8 patients received biventricular repair (see Tables 2 and 3). By looking at 3D prints in 8 patients, the feasibility of biventricular repair could be predicted with certainty before the definite repair and in two patients univentricular repair could be predicted with certainty before definite repair. In patient 6, conventional imaging showed that it was impossible to close the VSD with a patch and an ASO seemed impossible. However, 3D print showed that this was an option and allowed for biventricular repair. In two patients biventricular repair / univentricular / 1,5 repair could not be predicted with certainty. This was the case in patient 5 where the tricuspid straddling was not observed in the 3D print and the choice for univentricular repair had to be made during surgery. One 3D print was made in retrospect of the surgery (patient 1), if the 3D print had been made in advance it could have been predicted that the LV could not be tunneled to the Ao without compromising the TV ostium (which excluded biventricular repair).

**Table 2:** Definite biventricular procedures

Pt nr	Biventricular	Age during procedure* (months)*	Procedure
3	Biventricular	17	Tunnel LV-PA, ASO, patch closure 2nd large inlet VSD
6	Biventricular	3	Tunnel LV-PA, ASO, aortic arch repair
7	Biventricular	20	Tunnel LV-Ao
8	Biventricular	8	Tunnel LV-Ao, transannular RVOT patch
9	Biventricular	10	VSD enlargement, Nikaidoh
10	Biventricular	2	Tunnel LV-PA, ASO, relocation TV chords
12	Biventricular	3	Tunnel LV-Ao, ASO, relief RVOTO
13	Biventricular	13	Tunnel Lv-Ao, VSD enlargement, conduit RV-PA

*Ao* Aorta, *ASO* Arterial Switch Operation, *BCPA* Bidirectional Cavo Pulmonary Anastomosis, *DKS* Damus Kaye Stansel, *LV* Left Ventricle, *MBTS* Modified Black Taussig Shunt, *PA* Pulmonary artery, *PAB* Pulmonary Artery Banding, *RVOT(O)* Right Ventricular Outflow Tract (Obstruction), *TV* Tricuspid Valve

**Table 3:** Definite univentricular approaches

Pt nr	Univentricular	Age during procedure	Procedure
1	Univentricular	62	Tunnel LV-Ao not possible; tunnel LV-PA obstructs TV, Fontan
4	Univentricular	81	VSD too big for septation, Fontan
5	Univentricular	55	Straddling TV inhibits biventricular repair, Fontan
11	Univentricular	9	VSD too big for septation, BCPA DKS TV repair
2	Univentricular	32	Septation not possible, small LV; PAB only because of pulmonary hypertension

*Ao* Aorta, *ASO* Arterial Switch Operation, *BCPA* Bidirectional Cavo Pulmonary Anastomosis, *DKS* Damus Kaye Stansel, *LV* Left Ventricle, *MBTS* Modified Black Taussig Shunt, *PA* Pulmonary artery, *PAB* Pulmonary Artery Banding, *RVOT(O)* Right Ventricular Outflow Tract (Obstruction), *TV* Tricuspid Valve

With the help of 3D prints, in eight patients the exact surgical technique to accomplish biventricular repair could be predicted with high certainty preoperatively. In two univentricular patients, good predictions were able to be made and in two patients univentricular surgical techniques were not able to be predicted with certainty.

In addition to determining the surgical technique and predicting feasibility of biventricular, 3D prints allowed for better consultation with parents.

Tables 2 and 3 The definite surgical approach taken either biventricular of univentricular.

## Discussion

Surgical planning and determining biventricular or univentricular approach can be notoriously difficult in cDORV and 3D printing is a useful tool to overcome the limitations of conventional imaging [4]. A 3D print allows for spatial and mental simulation in surgical planning [1]. The hollow 3D models give detailed information on intracardiac and extracardiac structures [6].

The actual repair can be simulated after opening the 3D model. 3D prints can be used in discussing the diagnosis with both family/caretakers and other medical professionals and help to coordinate the surgical plan. Printed models can better identify suitable patients for biventricular repair and help in deciding upon the optimal surgical method [19]. Biventricular repair gives better post-operative outcomes and less reinterventions are necessary than with palliative univentricular repair [7]. As seen in Table 4, in 10 out of 12 patients (3D print of patient 1 was made in retrospect), 3D prints allowed for adequate prediction of surgical approach and technique.

**Table 4:** The adequacy of the value of the surgical predictions made based on the 3D print

Pt nr	Biventricular/Univentricular	Prediction value 3D print
1	Univentricular	In retrospect, based on 3D print a different surgical approached should have been taken
2	Univentricular	Adequately
3	Biventricular	Adequately
4	Univentricular	Adequately
5	Univentricular	Not adequately
6	Biventricular	Adequately
7	Biventricular	Adequately
8	Biventricular	Adequately
9	Biventricular	Adequately
10	Biventricular	Adequately
11	Univentricular	Not entirely adequately, but sufficient to exclude biventricular approach
12	Biventricular	Adequately
13	Biventricular	Adequately

### Limitations

Since the 3D prints used are made over a period of 12 years (2011–2023), the printing quality differs and not all could be used to practice the procedure as some are of hard plastic. Purchasing 3D-printed heart models is expensive which may form an obstacle for practical use [20]. The costs vary around 500 euros, depending on the type of the model [21]. Furthermore, tricuspid straddling can be missed when looking at a 3D print and hence should be closely looked at on the echocardiograph next to the print to avoid in surgery surprises. Internal 3D printing services in hospitals or virtual models of the heart could be considered as an alternative for 3D printed model service. We have analyzed internal production costs and each heart model can be produced with a cost under 86 euros [13]. Milano et al. [19] found that using virtual reality in DORV repair causes an increase in accuracy of selection of suited patients for biventricular repair to 95% in compared to 75% with cross-sectional imaging techniques (CT and MRI) [19]. Virtual reality is less expensive, and it takes less time to develop an image [22]. 3D models cannot clearly demonstrate the TV and its apparatus despite its importance in determining whether routing is possible. Currently in small pediatric printed heart models there is not yet a solution for this. However, in adult hearts work is being done to visualize AV valves in 3D models by merging data of echo and CT scan.

Virtual reality imaging also requires more technical expertise [23]. Another added value of 3D prints is that using 3D models allows for surgical simulation to practice retracting, cutting, suturing, and handling of the procedure [20].

### Conclusion

Planning surgery for cDORV patients and predicting the feasibility of biventricular repair is notoriously difficult. 3D printing can be used to predict the feasibility of biventricular repair with more accuracy. Additionally, 3D prints can predict the type of surgical technique to obtain biventricular repair better. Therefore, a 3D print is strongly recommended to be used in addition to conventional imaging in patients with Complex DORV.

## Data Availability

No datasets were generated or analysed during the current study.

## Appendix 1

### Previous Palliative Surgeries

See Table 5

**Table 5:** Previous palliative surgeries obtained from LUMC database

Pt nr	Previous palliative surgeries
1	PAB, BCPA, Atrial septectomy
2	2012 PAB and 2015 PAB loosening
3	Pab and coarctectomy
4	BCPA
5	MBTS right, atrial septectomy and MBTS left, later replaced by BCPA
6	Bilat PAB and ductal stent
7	PAB and coarctectomy
8	MBTS × 2
9	MBTS
10	-
11	Aortic arch repair and PAB
12	-
13	MBTS

*Ao* Aorta, *AV* Atrioventricular, *BCPA* Bidirectional Cavo Pulmonary Anastomosis, *DORV* Double Outlet Right Ventricle, *LV* Left Ventricle, *MBTS* Modified Black Taussig Shunt, *NRA* Normally Related Arteries, *PA* Pulmonary artery, *PAB* Pulmonary Artery Banding, *PS* Pulmonary Stenosis, *RV* Right Ventricle, *TGA* Transposition of the Great Arteries (‘TGA position’), *TV* Tricuspid Valve, *VCI* Vena Cava Inferior, *VCS* Vena Cava Superior, *VSD* Ventricular Septal Defect

## Appendix 2

### Raw Patient Data and Characteristics

**Table:**

Patient nr	1	2	3	4	5	6
Age during surgery	*5y2m*	*2y8m*	*1y5m*	*6y9m*	*4y7m*	*0y3m*
Diagnostics	DORV TGA, VSD non-committed	DORV NRA, VSD multiple, LV hypoplasia	DORV TGA, VSD multiple, coarctat ion	DORV Ao left to PA, dextrocardia, AV discordance, VSD inlet large, straddling TV	DORV TGA, juxtaposition auricles, double VCS, criss-cross AV connection s, VSD multiple, straddling TV, PS	DORV TGA, VSD non- committed, aortic arch obstruction,
Biventricular/ univentricular	Univentricular	Univentricular	Biventricular	Univentricular	Univentricular	Biventricular
ASD	Yes	Yes	Yes	No	Yes	Yes
Outflow with tract obstruction	No	No	No	No	Yes PS	No
Perimembranous/ n on-perimembranous VSD	Large perimembranous inlet	Perimembra nous	Perimembranous outlet and muscular inlet	Perimembranous	Perimembranous outlet and inlet	One perimembra nous and one muscular
Location VSD and relation to tricuspid valve	Non-committed	Multiple; apical and mid-musculus	multiple: perimembranous, outlet musculus and inlet	Inlet and outlet	Perimembranous and muscular VSD	Non- committed VSD

**Table:**

Patient nr	7	8	9	10	11	12	13
Age during surgery	1y8m	0y8m	0y10m	0y2m	0y9m	0y3m	1y1m
Diagnostics	DORV NRA, VSD non-committed	DORV NRA, left isomerism, vena azygos continuation of VCI, PS	DORV TGA, VSD Subpulmonary, pulmonary stenosis	DORV TGA, VSD outlet, straddling TV	DORV TGA, VSD large non-committed, RV hypoplasia	DORV TGA, VSD subpulmonary, PS subvalvar (fibrous tissue)	DORV, TGA
Biventricular/ univentricular	Biventricular	Biventricular	Biventricular	Biventricular	Univentricular	Biventricular	Biventricular
ASD	Yes	Yes	Yes	Yes	Yes	Yes	No
Outflow with tract obstruction	No	yes pulmonary outflow tract obstruction,	Yes PS	No	No	Yes, subpulmonary obstruction by accessory fibrous tissue underneath pulmonary valve	RVOT
Perimembranous/non- perimembranous VSD	Perimembranous inlet and outlet	Perimembranous	Perimembranous	Perimembranous outlet	Perimembranous inlet and outlet	perimembranous	Perimembranous
Location VSD and relation to tricuspid valve	Non-committed	Subaortic	Subpulmonary	Subpulmonary	Outlet and inlet	Sub pulmonary	Non-committed

**Table:**

Patient nr	1	2	3	4	5	6	
Aortic abnormalities	*No*	*No*	*Yes; aortic arch hypoplasia and coarctation*	*No*	*No*	*Yes; aortic arch hypoplasia and coarctation*	
PA abnormalities	No	No	No	PS	PS	No	
TGA	Yes: aorta in front right	No	Yes; aorta and PA side to side	No	Yes	Yes	
Great arterial relationships	Normal	Normal	Ao and PA side to side	Ao left in front and PA right in the back	Ao in front of PA	Ao in front of PA	
Coronary anatomy	2LCXR	*1R2LCX*	1RLCX	2RLCX	1R2LCX	1L2CXR	
Persistent ductus arteriosus	No	yes: closed with plug after first PAB	Yes	No	Yes	Yes	
Venous malformations	No	Yes: Double VCS	No	No	yes: double VCS and juxtaposition of both auricles	no	
Previous palliative surgeries	PAB, BCPA, Atrial septectomy	2012 PAB and 2015 PAB loosening	PAB and coarctectomy	BCPA	MBTS right, atrial septectomy and MBTS left, later replaced by BCPA	Bilat PAB and ductal stent	
Procedure	Tunnel LV-Ao not possible; tunnel LV-PA obstructs TV, Fontan	Septation not possible, small LV; PAB only because of pulmonary hypertension	Tunnel LV-PA, ASO, patch closure 2nd large inlet VSD	VSD too big for septation, Fontan	Straddling TV inhibits biventricular repair, Fontan	Tunnel LV- PA, ASO, aortic arch repair	
Certainty surgical plan	In retrospect different procedure	Good	Good	Good	Not good	Good	
Feasibility to close VSD with patch	No	No	Yes	No	No	Yes	
Straddling tricuspid and mitral valve	No	No	No	No	yes: criss-cross AV connection	No	
Size of the tricuspid and mitral valve	Normal	Too small mitral valve	Normal	Normal	Normal	Normal	
Size VSD	Large VSD	different sizes VSD	different t sizes VSD	large VSD	large VSD	normal	
Size of both ventricles	Normal	LV too small	Normal	RV too small	Normal	Normal	
Systemic venous connections	Normal	Double VCS	Normal	Normal	Double VCS	Normal	
Muscular infundibulum present or not	Yes; subaortic	Yes; subaortic	Yes; subaortic	Yes; subaortic	Yes; subaortic	Yes; subaortic	
Timing 3D print; made before or after definite procedure	The 3D print was made after Glenn shunt in 2012 First surgery without 3D print where it was seen that biventricular repair was not possible. This could have been seen beforehand if there would have been a 3D print before. This would have also allowed for better consultation parents. It would have led to shorter surgical planning. The LV could not be tunneled to Ao without making TV too small	3D print made before second PAB, led to the decision to only loosen first PAB, this could have not been seen on conventional 2D images	3D print before repair, allowed for biventricular repair, without 3D print it would have been univentricular repair	3D print made before surgeries; it was impossible to do surgical repair	Print made before Glenn procedure; did not help because the tricuspid straddling was not seen in the 3D print, the choice for univentricular repair had to be made during the surgery	3D print made before; allowed biventricular repair; first closing with patch and ASO seemed impossible, but 3D print showed that it was an option	

**Table:**

Patient nr	7	8	9	10	11	12	13
Aortic abnormalities	Yes: arch coarctation	No	No	No	Coarctation	No	No
PA abnormalities	No	PS	PS	No	No	No	No
TGA	No	No	Yes	Yes	No	Yes	Yes
Great arterial relationships	Normal	Ao in PA side to side	Ao in front of PA	side to side	Ao right in front of PA	Ao in front of PA	Aorta right anterior to PA
Coronary anatomy	*1R2LCX*	2LCXR	1L2R	1R2LCX	1R2LCX	1L2R	1R2LCX
Persistent ductus arteriosus	No	No	No	No	Yes	No	No
Venous malformations	No	left isomerism, vena azygos continuation of VCI	No	No	No	No	Azygous continuation VCI
Previous palliative surgeries	PAB and coarctectomy	MBTS × 2	MBTS	No	Aortic arch repair and PAB	No	MBTS
Procedure	Tunnel LV-Ao	Tunnel LV-Ao, transannular RVOT patch	VSD enlargement, Nikaidoh	Tunnel LV- PA, ASO, relocation TV chords	VSD too big for septation , BCPA DKS TV repair	Tunnel LV-Ao, ASO, relief RVOTO	Tunnel Lv-Ao, VSD enlargement, conduit RV-PA
Certainty surgical plan	Good	Good	Good	Good	unsure; but good to exclude biventricular repair	Good	Good
Feasibility to close VSD with patch	Yes	Yes	Yes	Yes	No	Yes	Yes
Straddling tricuspid and mitral valve	No	No	No	yes: straddling TV	No	No	No
Size of the tricuspid and mitral valve	Normal	Normal	Normal	Normal	Normal	Normal	Normal
Size VSD	Large VSD	Large VSD	Small: enlarged during Nikaidoh	Large VSD	Large VSD	Large VSD	Small
Size of both ventricles	Normal	Normal	Normal	Normal	RV smaller than LV	RV smaller than LV	Normal
Systemic venous connections	Normal	Vena azygos continuation of VCI	Normal	Normal	Normal	Normal	Azygous continuation VCI to VCS
Muscular infundibulum present or not	Yes; subaortic	Yes; subaortic	Yes; subaortic	Aorta on muscular conus with a 30 degrees angle	Yes; subaortic	Yes; subaortic	Yes: RVOT
Timing 3D print; made before or after definite procedure	3D print made before which allowed the decision for biventricular repair	3D print before repair, this allowed for decision biventricular repair, without 3D print a univentricular repair would have been done	3D print before which allowed for biventricular repair	3D print before which allowed for biventricular repair and helped in determining surgical technique: ASO and Tunnel Lv- PA	3D print before procedure to decide on biventricular or 1.5ventri cular repair Conclusion was able to be made to choose univentricular or 1.5ventri cular repair based on 3D print. First septating seemed	3D prints before: allowed to determine biventricular repair and surgical technique	Made before definitive procedure

## Appendix 3

### Considerations Based on the 3D Print per Patient

Patient 1: The 3D print was made after the Glenn shunt in 2012. In this first surgery without 3D print it was seen that biventricular repair was not possible. The LV could not be tunneled to Ao without making the TV too small. This could have been observed beforehand if a 3D print had been available. This would have also allowed for better consultation of the parents and would have led to shorter surgical planning.

Patient 2: The 3D print was made before the second PAB. This led to the decision to only loosen first PAB which could have not been seen on conventional 2D images.

Patient 3: The 3D print before definite repair allowed for the decision on biventricular repair. Without the 3D print, the decision would have been univentricular repair.

Patient 4: The 3D print was made before surgeries, this showed that it was impossible to do surgical repair.

Patient 5: The 3D print was made before the Glenn procedure which did not give enough adequate information because the tricuspid straddling was not seen in the 3D print. The choice for univentricular repair had to be made during the surgery.

Patient 6: The 3D print made beforehand allowed biventricular repair. At first closing VSD with patch and ASO seemed impossible, but the 3D print showed that it was an option.

Patient 7: The 3D print was made before which allowed the decision for biventricular repair.

Patient 8: The 3D print before repair allowed for decision on biventricular repair, without 3D print an univentricular repair would have been done.

Patient 9: The 3D print was made before the definite repair which allowed for the decision of biventricular repair.

Patient 10: The 3D print was made before which allowed for biventricular repair and helped in determining surgical technique: Arterial Switch Operation (ASO) and Tunnel Lv-PA.

Patient 11: The 3D print was made before the procedure to decide on biventricular or 1.5 ventricular repair. This showed that the best option was univentricular or 1.5 ventricular repair. This decision was made because first septating seemed feasible before the print was made however the VSD was too large which would lead to a too small RV.

Patient 12: The 3D print before allowed to determine biventricular repair and surgical technique.

Patient 13: The 3D print was made before which allowed for biventricular repair and helped in determining surgical technique. The 3D print showed that enlargement of VSD was necessary.

## Abbreviations

Ao: Aorta
ASO: Arterial switch operation
AV: Atrioventricular
BCPA: Bidirectional cavo pulmonary anastomosis
DKS: Damus kaye stansel
(c)DORV: (Complex) Double outlet right ventricle
LV: Left ventricle
LVOT(O): Left ventricular outflow tract (Obstruction)
MBTS: Modified black taussig stunt
NRA: Normally related arteries
PA: Pulmonary artery
PAB: Pulmonary artery banding
PS: Pulmonary stenosis
RV: Right ventricle
RVOT(O): Right ventricular outflow tract (Obstruction)
TGA: Transposition of the great arteries
TV: Tricuspid valve
VCI: Vena cava inferior
VCS: Vena cava superior
VSD: Ventricular septal defect
